# The state of the human coding gene catalogues

**DOI:** 10.1093/database/baaf045

**Published:** 2025-09-24

**Authors:** Miguel Maquedano, Daniel Cerdán-Vélez, Michael L Tress

**Affiliations:** Bioinformatics Unit, Spanish National Cancer Research Centre (CNIO), Calle Melchor Fernandez Almagro, 3, 28029 Madrid, Spain; Bioinformatics Unit, Spanish National Cancer Research Centre (CNIO), Calle Melchor Fernandez Almagro, 3, 28029 Madrid, Spain; Bioinformatics Unit, Spanish National Cancer Research Centre (CNIO), Calle Melchor Fernandez Almagro, 3, 28029 Madrid, Spain

## Abstract

In 2018, we analysed the three main repositories for the human proteome: Ensembl/GENCODE, RefSeq, and UniProtKB. At that time the three gene sets disagreed on the coding status of one of every eight annotated coding genes, and our results suggested that as many as 4234 of these genes might not be correctly classified. Here, we have repeated the analysis with updated versions of the three reference gene sets. Superficially, little appears to have changed. The three sets annotate 21 871 coding genes, slightly fewer than previously, and still disagree on the status of 2603 annotated genes, almost one in eight. However, we show that collaborations between the three reference gene sets have led to greater consensus. Reference catalogues have agreed on the coding status of another 249 genes since the last analysis while at least 700 genes have been reclassified. We still find that there are >2000 coding genes with at least one potential non-coding feature to indicate that they may not be coding genes. This includes a large majority of the 2603 genes for which annotators do not agree on coding status. In total, we believe that as many as 3000 genes may be misclassified as coding and could be annotated as non-coding genes, pseudogenes, or cancer antigens.

## Introduction

Since the initial release of the human genome sequence almost a quarter of a century ago [1, 2], annotators have been carrying out a detailed curation of the coding and non-coding genes found in the genome. The analysis of the protein coding gene complement has been implemented principally by three groups of researchers. The Ensembl/GENCODE [3, 4] and RefSeq [5] reference sets are based on genomic coordinates, and the UniProtKB [6] proteome is based on the recorded proteins

Many of the genes in the three reference sets emerged from one the genetic sequence collections that make up the International Nucleotide Sequence Database Collaboration [7], while others began life as automatic gene predictions or were provided by outside research groups. However, coding genes are now only added to the reference gene sets when there is sufficient experimental or conservation evidence to support their annotation. These genes, and their related transcripts and protein isoforms, can also undergo changes in status as existing annotations are revised. Many genes are reclassified as a result of the annotators adjusting to the available evidence, and some of the more difficult to discriminate genes may be reclassified more than once. This has occurred with the pseudogene *WASH6P*, e.g. [8].

Before the human genome was sequenced, estimates for the number of human coding genes were between 25 000 and 40 000, but since then a number of large-scale analyses have revised the estimates downwards to between 19 000 and 22 000 genes [9–14], with the current number of annotated coding genes in the two coordinate-based reference sets much closer to the lower of the two figures. The estimate of coding genes in the Ensembl/GENCODE gene set has been steadily increasing in recent releases because of a push to find evidence for small open reading frames (ORFs) missed by annotators [15].

Annotators often add protein coding genes based on experimental evidence from external research groups. Most research groups have been focused on coding gene discovery, largely because it is more rewarding to find new coding genes than it is to demonstrate that predicted coding genes do not produce proteins, but also because it is not possible to demonstrate that an ORF does not code for a protein. Only a few groups have attempted to predict which coding genes might have been misclassified by annotators [9, 10, 12, 14]. Clamp *et al*. [9] showed that most annotated novel ORFs resembled non-coding RNA rather than coding genes and estimated that there were just 20 500 human coding genes, while Church *et al*. [10] compared the mouse and human gene sets and predicted that the number of human coding genes was below 20 000. Many coding genes were reclassified as not coding after these two analyses.

We have published two previous analyses in which we attempted to uncover genes that were likely to have been misclassified as coding [12, 14]. We used a series of indicators, called potential non-coding features, to classify genes as potential non-coding [12, 14]. We found that genes with potential non-coding features had almost no peptide support in large-scale proteomics analyses, and many of the potential non-coding genes we identified have since been reclassified by manual curators. In 2014, we flagged 2001 potential non-coding genes in the GENCODE v12 gene set [12]. Almost half of these genes (908) were withdrawn or reclassified from the human reference set by the expert curators in GENCODE. In 2018, using the GENCODE v24 gene set, we labelled another 2278 genes as potential non-coding [14]. Since then, 379 of these coding genes have been reclassified as not coding.

In the second analysis, we merged the Ensembl/GENCODE, RefSeq, and UniProtKB gene sets and found that the union of the three reference sets annotated 22 210 coding genes [14]. We showed that one in eight annotated coding genes were not regarded as coding by all three curation groups [14]. As a result of the second analysis, curators of the three reference sets set up joint projects such as MANE [16] to converge on an agreed set of coding genes and transcripts.

Here, we have updated our analysis of the human coding gene catalogue. We merged the most recent Ensembl/GENCODE, RefSeq, and UniProtKB gene sets and found that there has been convergence between the three databases, but that there are still thousands of genes that the three databases do not agree on. We also tag >2000 potential non-coding genes and many of these genes are likely to have been misclassified as coding.

## Materials and methods

### The Ensembl/gencode reference set

We downloaded the coding genes from the Ensembl 111/GENCODE v45 reference set from the BioMart tool [3], restricting the list to protein coding genes in the 24 distinct human chromosomes and the mitochondrial chromosome. At the same time, we downloaded the equivalent gene/protein identifications that BioMart had stored for UniProtKB and RefSeq. We downloaded 20 444 Ensembl/GENCODE coding genes.

### The UniProtKB reference proteome

We downloaded those reviewed UniProtKB [6] entries that were tagged by UniProtKB as belonging to the UP000005640 (human) proteome in the 27th March 2024 version of the UniProtKB database. Along with the list of proteins we downloaded, we included the HGNC gene names [17] and the Ensembl and RefSeq cross-references that were recorded by UniProtKB. After processing (see later) we ended up with 20 484 entries. UniProtKB has developed an automatic pipeline to annotate Ensembl/GENCODE genes directly into UniProtKB Trembl. Many of these are included in the UniProtKB human proteome, but we did not include these unreviewed entries in our analysis. More than two thirds of the UniProtKB Trembl sequences we left out were Immunoglobulin/T-cell receptor fragments or from readthrough genes.

### The RefSeq reference gene set

We downloaded the list of human protein coding genes from the NCBI gene database [5] at https://www.ncbi.nlm.nih.gov/datasets/gene/. This produced 20 441 coding genes. We selected only those in the GRCh38 assembly because RefSeq has already included predictions for the CHM13 assembly. Of these, seven were coding loci that covered the immunoglobulin and T-cell receptor fragment clusters. Individual immunoglobulin and T-cell receptor (IG/TR) fragments are defined as ‘other’ in the RefSeq genome. The final list of coding genes contained 19 950 coding genes.

### Merging the three sets

The sets were merged initially via their HGNC symbols and discrepancies were curated manually. Since the UniProtKB database is based on proteins rather than genes, UniProtKB had the most obvious discrepancies. Some genes had multiple UniProtKB entries (e.g. *TMPO*, which has entries for alternatively spliced proteins LAP2 alpha and LAP2 beta/gamma), while some UniProtKB protein-sequence identical entries pointed to multiple genes (e.g. entry Q9ULZ0, which maps to six ‘coding’ genes, *TP53TG3, TP53TG3B, TP53TG3C, TP53TG3D, TP53TG3E*, and *TP53TG3F*). Both sets of discrepancies had to be deduplicated so that each coding gene corresponded with a single UniProtKB entry. In addition, UniProtKB includes proteins that came from genes only present in scaffolds. These were removed.

Other corrections were made based on BioMart cross-references and UniProtKB ID mapping cross-references where necessary. In more complicated cases, we checked the coordinates of the Ensembl/GENCODE and RefSeq genes with the UCSC genome browser [18]. RefSeq and Ensembl did have different numbers of coding genes at the same locus in a small number of cases, e.g. the gene *ERCC6*, where Ensembl/GENCODE has a single gene and RefSeq two. In these cases, we merged one of the multiple genes and left the second one without a match in the other reference set.

### Determining coding status of non-intersection genes

For the 2603 coding genes outside of the intersection between the three sets (genes annotated as coding in just one or two of the three reference sets, but not in all three), we searched for the status of the gene in the reference set (or sets) in which it was not annotated as coding or the reason why it was not included as a coding gene. Categories included readthrough genes, pseudogenes, IG/TR fragments, antisense transcripts, lncRNA, intergenic genes, UTR ORF genes, intronic genes, and retroviral genes.

‘Readthrough’ genes are genes with transcripts that run from one gene to a neighbouring gene and in GENCODE [4] are tagged in the *gtf* file. The status of ‘pseudogene’ was applied if the coding genes overlapped a pseudogene in another reference set, or if the gene had an HGNC name that indicated that the entry was a pseudogene. The ‘IG/TR’ fragment gene status was applied to those genes that were tagged as immunoglobulin or T-cell receptor fragments in Ensembl/GENCODE, and to those that were clearly immunoglobulin or T-cell receptor fragments from the associated HGNC gene name in UniProtKB. ‘Antisense’ status was applied when transcripts were on the opposite strand of a coding gene or a pseudogene in the other reference set and close to or overlapping the UTR of those genes, or when the HGNC name indicated that the gene was antisense to another gene. Genes were labelled as lncRNA when they overlapped exons from lncRNA in the same sense in the other reference set or if the gene had an HGNC name that indicated that the entry was originally from a lncRNA gene.

The status ‘intergenic’ was reserved for those genes that did not overlap any other type of gene in the other reference set and that fell between annotated genes, while those tagged as ‘no coordinates’ were all from UniProtKB but did not map to the GRCh38 genome. ‘UTR ORF’ covered those coding genes that overlapped with UTR in another reference set, ‘intronic’ covered those coding genes that were found wholly within introns of genes in the other reference set, and artefact and ‘TEC’ (To be Experimentally Confirmed) covered those coding genes that were annotated as such in the equivalent region in the Ensembl/GENCODE reference set. Genes were labelled as ‘retroviral’ when they overlapped exons from retroviral genes in the other reference set or if the gene had an HGNC name that indicated that the entry was originally from a retrovirus.

### Potential non-coding features

Potential non-coding features were taken directly from the UniProtKB and/or the Ensembl/GENCODE annotations. The three exceptions were PhyloCSFMax, which is the standard PhyloCSF [19] score of the highest scoring exon that has a minimum 10 codons; gene family age, which is the normalized age of the most distant BLAST [20] hit from an APPRIS database [21] search against a limited list of RefSeq reference proteomes [5]; and ‘no protein features’, which applies to those genes that do not have functional residues, homology to protein structure or domains, cross-species conservation or transmembrane helices (all data from APPRIS modules), and in addition have a negative PhyloCSFMax score. Cut-offs used for potential non-coding features were −14 or lower for PhyloCSFMax and no homologues beyond catarrhini for APPRIS gene age.

### Peptide data

We also downloaded peptides from PeptideAtlas [22]. PeptideAtlas peptides had to be fully tryptic and have at least two observations. Peptides that mapped to more than one gene were left out of the analysis. Peptides were not filtered further, and we did not check for false positives. In this analysis we did not use the peptides as validation of coding potential or as a potential non-coding feature, merely as an indicator. Peptide data were available from the APPRIS database for all Ensembl/GENCODE genes.

### Human Protein Atlas data

The Human Protein Atlas (version 23.0) provides evidence for the transcription and translation of proteins in tissues. Translation is supported by western blots and antibodies. Annotated coding genes have their own summary page and eight different tabs relating to different tissues. We principally used the ‘Tissue’ tab to look for a summary of tissue-level transcription and translation. Transcription over a range of different tissues is supported by RNA-Seq from GTEx [23], Fantom [24], and data from the Human Protein Atlas itself [25]. Protein-level expression is not quantified. Instead, the score represents the estimated protein expression. The database also provides a reliability score (enhanced, supported, approved, or uncertain) for the antibody identification. We also used the Subcell tab, to look for reliable evidence of translation in tissues at the subcellular level, and the ‘Cancer’ tab to look for evidence of translation in cancer cells.

### Non-synonymous to synonymous ratios

To evaluate the ratio of non-synonymous to synonymous variants (NS/Syn ratio) as an indicator of potentially non-coding genes, we first processed variant data from GNOMAD genomes analysis [26] using the Variant Effect Predictor (VEP) tool [27]. We utilized the principal transcripts derived from the APPRIS database as the representative for each gene to maximize the coding potential [28, 29].

APPRIS principal transcripts [30] are chosen as the main transcript/isoform in coding genes based on the presence of protein structural and functional characteristics and cross-species conservation. They are one of the inputs to the MANE Select transcript selection process [16], and APPRIS and MANE agree on the main transcript in almost 95% of coding genes [28]. Comparisons between APPRIS principal and MANE Select transcripts show that both are excellent choices as the main transcript for coding genes. Their isoforms agree with the main proteomics isoform in 95% of coding genes [28], their exons are under purifying selection while exons in alternative transcripts are not [28], and reference transcripts chosen by MANE and APPRIS capture 99.9% of PubMed-supported pathogenic variants [29]. All analyses found that there were no significant differences between APPRIS principal and MANE Select transcripts [28, 29].

Following this, the VEP output was parsed into a format suitable for downstream analyses. Variants were filtered to include only those containing one of the four valid alleles (A, C, T, and G). The dataset was then further refined by retaining only the following types of variants: ‘start_lost’, ‘synonymous_variant’, ‘missense_variant’, ‘stop_gained’, ‘stop_lost’, ‘splice_donor_variant’, and ‘splice_acceptor_variant’. Additionally, ‘splice_region_variant’ was included if it co-occurred with other valid variant types, as these are counted according to their accompanying variant classification.

Frameshift variants were excluded from the analysis. We also removed variants lacking a Frequency_maxAF annotation (i.e. not included in the gnomAD database), and those with a maxAF of 0. Variants with a maxAF >0.005 were considered rare for the posterior analyses.

To assess the NS/Syn ratios across rare and common alleles in different subsets of potentially non-coding genes, we used just the missense and synonymous variants.

Potential non-coding features were defined from these NS/Syn ratios. If the NS/Syn ratio for the subset of genes was >2.0 in both common and rare alleles and the difference between the two sets was <0.3, the feature was determined to be potential non-coding. We defined seven potential non-coding features this way and added the readthrough tag as an eighth potential non-coding feature. Readthrough genes appear to be partly under purifying selection because most of their exons overlap known coding exons.

## Results

**Figure 1.**
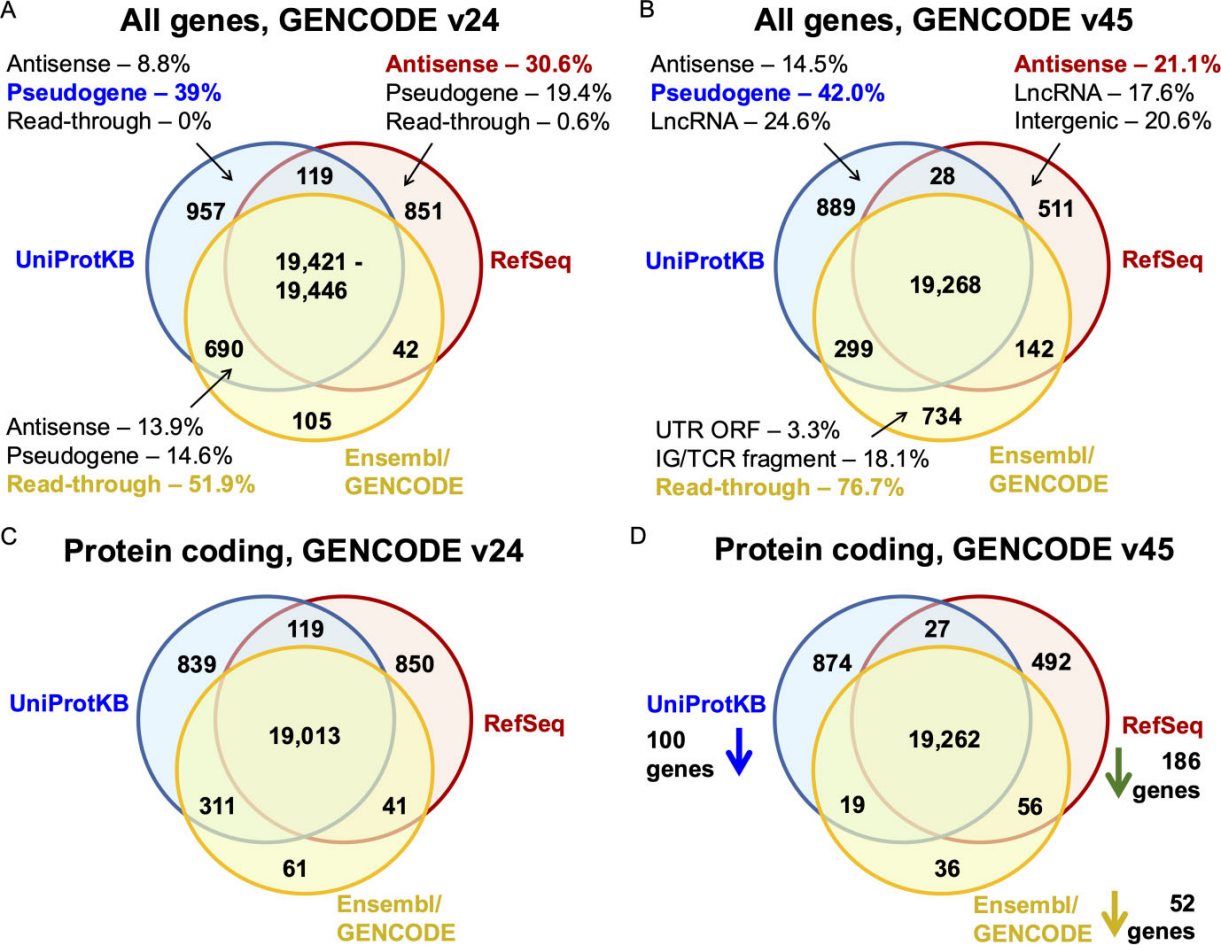
Reference coding genes contemporary to the GENCODE v24 and v45 sets. Coding genes in the merged sets of UniProtKB, RefSeq, and Ensembl/GENCODE (see the ‘Materials and methods’ section). In panel A, the count of all genes classified as coding in each of the three reference databases and the intersection between them at the time of release of the GENCODE v24 reference set [14]. In panel B, the count of all genes classified as coding in each of the three reference databases coinciding with the release of the GENCODE v45 reference set. Panel C shows the counts for the merge of the three annotations contemporary to the GENCODE v24 reference set excluding all readthrough genes and immunoglobulin and T-cell receptor fragments. In panel D, the same count, but at the time of the GENCODE v45 reference set, showing the net reduction of gene count in each of the three reference databases.

There were 21 871 coding genes in this merge of the three reference sets (Supplementary Table 1), slightly fewer than the 22 210 we counted in the previous analysis [14]. The distribution of coding genes between the three reference sets in the two merges can be seen in Fig. 1A and B. Of the 21 871 coding genes, 19 268 genes are predicted to be coding by all three sets of annotators, which means there are now 178 fewer genes agreed upon by all three databases with respect to our previous analysis. The three reference annotations fail to agree on the coding status of 2603 genes, so almost 12% of annotated coding genes still have different status in at least one of the three reference sets.

For the 2603 coding genes outside of the intersection between the three sets, we searched for the status of the gene in the reference set (or sets) in which it was not annotated as coding or the reason why it was not annotated as coding. The most common explanations as to why these genes were not annotated as coding were that they were readthrough genes (669), were annotated or tagged as pseudogenes (484), were annotated as IG/TR fragments (429), were antisense to a coding gene (239), were annotated as lncRNA (217), or were retrovirus-derived (72). Genes that had no equivalent in the other databases were tagged as intergenic (114), UTR ORF (90), or intronic (84) depending on their relative position. Further details on the categories are listed in the ‘Materials and methods**’** section.

Readthrough genes, a class of genes in which all transcripts are readthrough transcripts, make up a quarter (25.7%) of the genes that have different status in at least one of the three reference sets. Almost one in five genes with disputed coding status (18.6%) are annotated as pseudogenes by at least one of the other databases. For coding genes annotated by just one of the three databases, we have detailed the most common reasons for the discrepancies with the other two reference sets (Fig. 1A and B). For example, >40% of UniProtKB unique coding genes are annotated as pseudogenes in other gene sets, and three quarters of Ensembl/GENCODE unique genes are readthrough genes. Many RefSeq unique genes are likely non-coding, with antisense and LncRNA labels making up more than a third of differences.

### A merge without readthrough genes or immunoglobulin fragments

A sixth of the coding genes not agreed on by all gene annotators were immunoglobulin or T-cell receptor fragments. Even though T-cell receptor and immunoglobulin fragments produce protein regions, they are not always considered to be true protein coding genes. UniProtKB is based on protein sequences, so also includes representative whole immunoglobulin sequences, which RefSeq and Ensembl/GENCODE cannot annotate. In addition, RefSeq annotates these gene fragments as ‘other’ rather than coding and annotates seven protein coding loci in their place. Much of the decrease in RefSeq gene numbers since the 2018 analysis [14] is down to this technicality. This meant that many of these genes moved from the intersection in 2018 to the joint Ensembl/GENCODE and UniProtKB set in this analysis.

In the previous analysis, we included all the UniProtKB human proteome entries, including the non-reviewed entries. This time we left out the unreviewed sequences out of the merge. The unreviewed entries were almost entirely proteins from readthrough genes and T-cell receptor and immunoglobulin fragments, which pushed those genes still annotated as coding out of the agreement between UniProtKB and GENCODE in the 2018 comparison and into the Ensembl/GENCODE only category in this analysis (Fig. 1A and B).

Given that immunoglobulin and T-cell receptor fragments and readthrough genes are not treated in the same way by the three groups of curators, we carried out a comparison between the 2018 and current merges leaving out both sets of genes. Removing immunoglobulin and T-cell receptor fragments and readthrough genes from the comparison ought to provide a much better perspective of the effects of the Ensembl/GENCODE bilateral collaborations on the three reference sets.

When T-cell receptor and immunoglobulin fragments and readthrough genes are set aside from the comparison with the 2018 analysis [14], it becomes clear that all three reference sets actually annotate substantially fewer coding genes than in 2018 (Fig. 1C and D). At the same time, they agree on 249 more genes. In fact, there are 717 fewer coding genes outside of the intersection than there were in the 2018 analysis, which means 32.3% fewer disagreements between the three sets. These 717 genes have all been merged or reclassified.

What also stands out is that almost all the coding genes annotated by Ensembl/GENCODE (99.5%) are in agreement with the other two databases. If readthrough genes and T-cell receptor and immunoglobulin fragments are not coding, Ensembl/GENCODE has just 112 coding genes that are not part of both the other two reference sets (Fig. 1C and D). A merge without readthrough genes is not just a theoretical exercise, the Ensembl/GENCODE curators plan to reclassify readthrough genes as not coding. When they do this, many of the disagreements with the other two databases will disappear.

### Potential non-coding features for GENCODE v45 genes

In our previous analysis of the state of the human proteome [14], we defined 16 potential non-coding features. These were drawn from the UniProtKB and Ensembl annotations and from GENCODE consortium tools [19, 21]. We used these potential non-coding features to tag 2278 Ensembl/GENCODE genes as potential non-coding. Coding genes with one or more of these 16 features had very few peptides in large-scale tissue-based proteomics experiments [14]. They also had significantly lower transcript expression, many more copy number variants, twice as many potential high-impact variants and much higher synonymous to non-synonymous rates than other coding genes [14]. We theorized that many of these potential non-coding genes may not be *bona fide* coding genes.

For this paper, we used NS/Syn ratios of Ensembl/GENCODE genes to select potential non-coding features (see the ‘Materials and methods’ section for details on how the NS/Syn ratios were calculated). We classed a feature as potential non-coding if the difference between the common and rare NS/Syn ratios for genes with the feature was <0.3, and if the common and rare NS/Syn ratios were both >2.0. As a control, coding genes that had peptide support in PeptideAtlas had a rare NS/Syn ratio of 1.96 and a common NS/Syn ratio of 0.9, so are clearly under selection pressure.

**Figure 2.**
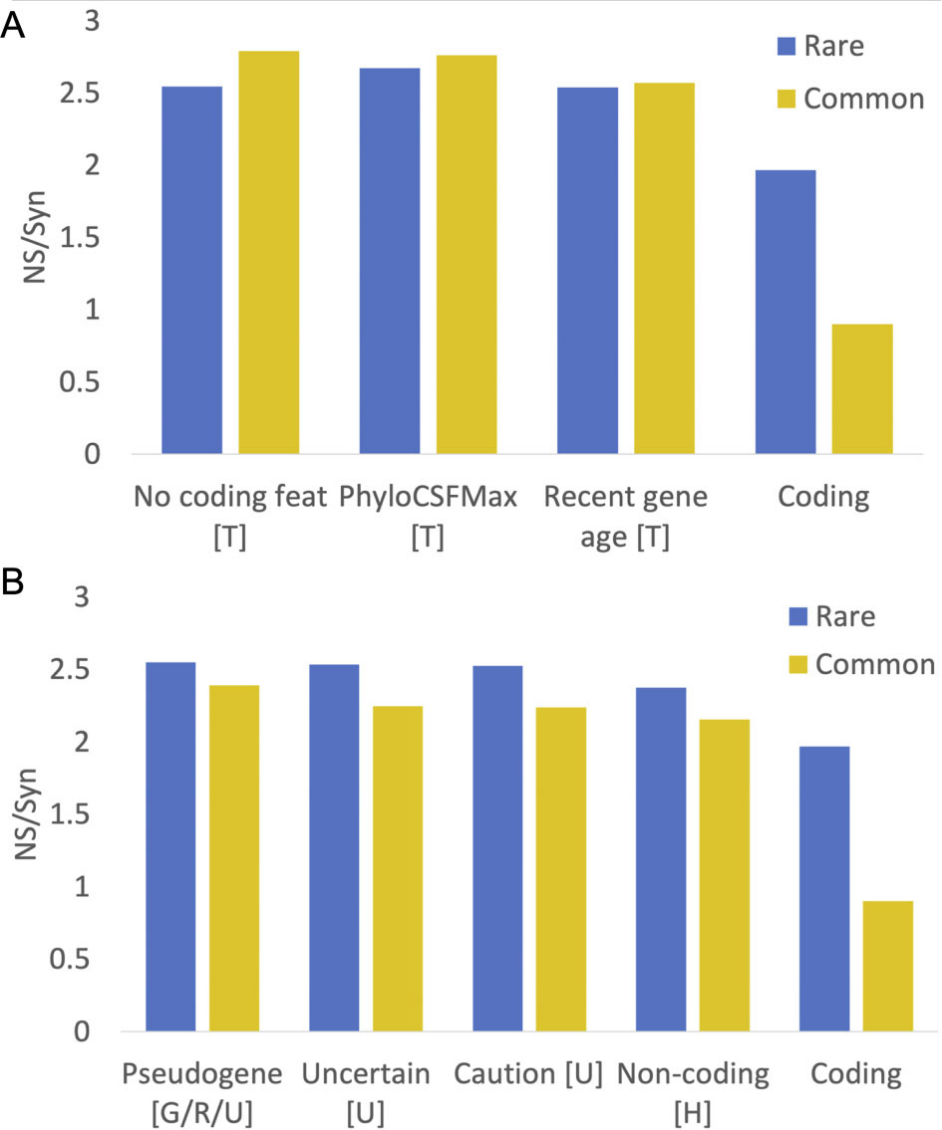
NS/Syn ratios for potential non-coding features in Ensembl/GENCODE. Panel A shows the calculated NS/Syn ratios for the three potential non-coding features that are based on a lack of protein-like conservation and are calculated with GENCODE tools PhyloCSF and APPRIS. These features, marked with a ‘T’, were calculated in-house from GENCODE data and are detailed in the ‘Materials and methods’ section. Panel B shows NS/Syn ratios for features taken directly from annotation by manual curators. Features with a ‘U’ came directly from the UniProtKB database, while features with a ‘G/R/U’ were taken from the GENCODE, RefSeq, and UniProtKB annotations. Features marked with an ‘H’ were taken from the HGNC name. In both panels, the NS/Syn ratios for coding genes supported by PeptideAtlas peptides are included as a control.

Just seven features met these criteria (Fig. 2): UniProtKB cautions (‘dubious CDS’ and ‘possible pseudogene’), UniProtKB ‘uncertain’ evidence code, the word ‘pseudogene’ in the protein description, recent gene family age (calculated through the APPRIS database [20]), poor PhyloCSF conservation [18], lack of protein features (calculated from both APPRIS and PhyloCSF scores), and the HGNC gene name [16]. Four of these features were maintained from the previous analysis, two are modified, and one is new. Several potential non-coding coding features from the previous analysis were excluded because they either tag minimal numbers of genes or have NS/Syn ratios that did not meet the criteria for this paper. Two of the main potential non-coding features from the previous analysis, UniProtKB ‘homology’ and ‘predicted’ evidence codes do have high NS/Syn ratios in both rare and common alleles, but ratios for common alleles were >0.3 points lower than the equivalent NS/Syn ratios for rare alleles (Supplementary Fig. 1). Not including these two features as potential non-coding is the main reason why there are many fewer potential non-coding genes in this analysis. Nevertheless, the high NS/Syn ratios for these two features suggest that many of these genes are not under selective pressure either.

We added readthrough genes to the final list of potential non-coding features even though the common variant NS/Syn ratio of this feature was below the threshold. Readthrough genes will inevitably have lower common variant NS/Syn ratios because a large majority of their exons overlap coding exons in the same frame. The final list of potential non-coding features is shown in Table 1. With these eight potential non-coding features, we tagged 1121 genes in GENCODE v45 (5.5%) as potential non-coding.

**Table 1.** The eight potential non-coding features, the number of genes tagged by each feature, and the number of those genes that had at least two supporting peptides in the PeptideAtlas database^a^

Potential non-coding feature	Genes	Peptide support
Recent gene age [T]	241	19
No protein features [T]	118	20
Readthrough gene [G/R]	655	0
PhyloCSF maximum [T]	98	10
Caution note [U]	91	24
Uncertain evidence [U]	83	22
Described pseudogene [G/R/U]	54	19
Non-coding name [H]	38	5

a The letter in brackets refers to the source of the feature, GENCODE tools APPRIS and PhyloCSF (T), the GENCODE annotation (G), RefSeq (R), UniProtKB (U), and HGNC (H).

### Protein evidence for non-coding GENCODE v45 potential non-coding genes

Peptide support was calculated for each gene from the peptides in the PeptideAtlas database [22]. Table 1 shows the number of genes with each potential non-coding feature that are supported by at least two tryptic peptides in PeptideAtlas. PeptideAtlas has peptide data from 3160 mass spectrometry experiments, so it has a much higher coverage than the experiments we used in our previous analysis [14]. As a comparison, PeptideAtlas provides peptide support for 89.2% of the GENCODE v45 genes not tagged as potential non-coding.

The disadvantage of using such a large source of peptide support is that we cannot validate the spectra, so a proportion of the identifications are almost certainly false positives. Given the similarity to known coding genes, the features that are most likely to have false-positive matches are those that involve pseudogenes. It is not surprising then that one of these features has the highest proportion of peptide support (pseudogene description, 35.2%). Readthrough genes have the least peptide support—just one readthrough transcript was supported by a single peptide in PeptideAtlas. Besides readthrough genes, those groups with the least evidence of cross-species conservation (recent gene age, 7.9%, and poor PhyloCSF, 10.2%) have the least peptide support.

### Potential non-coding genes outside of GENCODE

**Figure 3.**
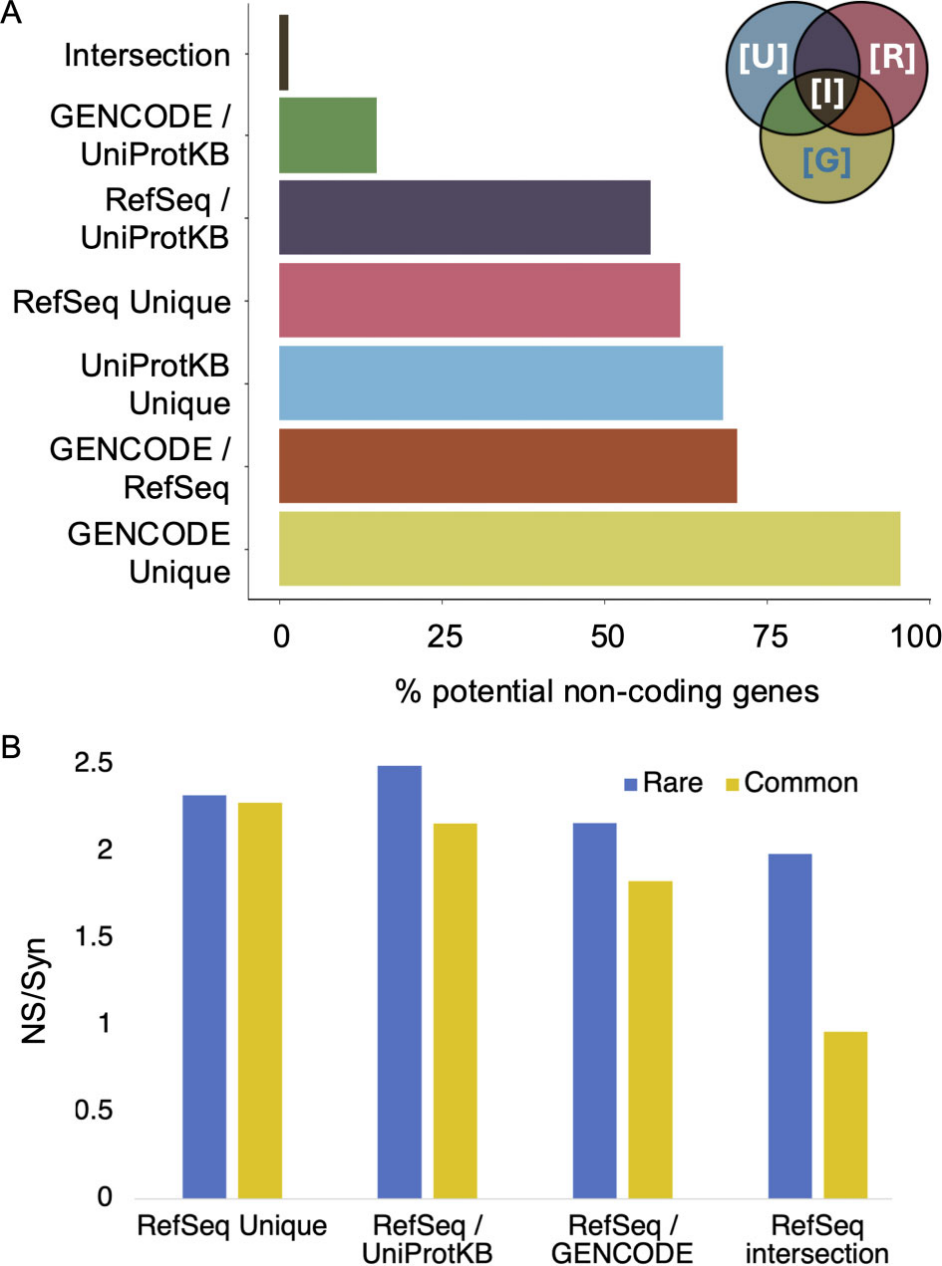
Potential non-coding genes beyond the intersection and RefSeq NS/Syn ratios. (A) The percentage of the genes in different gene sets that are tagged as potential non-coding genes. The genes in each set are those in Fig. 1B, and the bars are coloured as per the Venn diagram in the legend. In the legend, ‘[G]’ marks the Ensembl/GENCODE genes, ‘[R]’ marks all RefSeq genes, and ‘[U]’ indicates all UniProtKB genes. ‘I’ indicates the intersection. The ‘GENCODE/UniProtKB’ section has fewer potential non-coding genes because many of them are immunoglobulin/T-cell receptor fragments. The ‘GENCODE Unique’ section has proportionally more potential non-coding genes because almost all genes are readthrough genes. (B) The NS/Syn ratios calculated for predicted RefSeq coding genes. Common alleles in yellow, rare in blue. ‘RefSeq Unique’ are those RefSeq coding genes unique to RefSeq, ‘RefSeq/UniProtKB’ are RefSeq coding genes annotated by UniProtKB and not GENCODE, ‘RefSeq/GENCODE’ are RefSeq coding genes annotated by GENCODE and not UniProtKB, and ‘RefSeq intersection’ are RefSeq coding genes in the intersection of the three sets.

Over all three curated sets, 2059 of the 21 871 coding genes were tagged as potential non-coding (9.4%). Genes that had different status in the three genes sets had a much higher proportion of potential non-coding features (Fig. 3); 1784 of genes that were not annotated as coding in all three reference sets (68.5%) were predicted to be potentially non-coding, while among genes annotated as coding in all three gene sets just 275 had potential non-coding features (1.4%).

The potential non-coding features were designed for Ensembl/GENCODE genes, so not all of the features apply to the other two gene sets. Despite this, 668 of 1216 of UniProtKB proteins outside of the intersection (54.9%) were predicted to be products of potential non-coding genes and almost two thirds of the RefSeq genes not in the intersection (63.1%) were tagged with potential non-coding features. Just three of the eight potential non-coding features were applicable to RefSeq genes.

We could also calculate NS/Syn ratios for RefSeq genes and this allowed us to evaluate the functional relevance of genes unique to RefSeq. There was clearly a marked difference between the 19 269 RefSeq genes in the intersection and those outside. Over the 681 genes that did not agree with at least one of the other two databases, the NS/Syn ratio for rare alleles was 2.31, while the NS/Syn ratio for common alleles was 2.24 (Fig. 3B). This strongly suggests that the vast majority of RefSeq genes outside of the intersection are not under purifying selection. NS/Syn ratios for common alleles are slightly lower for the 170 genes annotated in conjunction with Ensembl/GENCODE or UniProtKB (Fig. 3B).

### Recently evolved potential non-coding genes

Of the 275 potential non-coding genes in the intersection some are clearly coding. For example, the *MGAM2* and *SRGAP2C* gene products have >50 peptides each in PeptideAtlas. These genes are only tagged as potential non-coding because UniProtKB still includes the word ‘pseudogene’ in their description. When UniProtKB updates its annotations, these genes will no longer be potential non-coding. At the same time, there are numerous potential non-coding genes in the intersection that are clearly not coding. To start with 6 readthrough genes are annotated as coding in all three reference sets, and none of these are *bona fide* protein coding genes.

Besides the 6 readthrough genes, there are other clear examples of potential non-coding genes in the intersection that have little or no supporting information. *HEPN1* is one, a predicted coding gene that is antisense to *HEPACAM* on chromosome 11. *HEPACAM* was first found in the liver [31] but is predominantly expressed in the brain. Curiously, *HEPN1* was identified by the same group before they described *HEPACAM* [32], but at no point was it established that *HEPN1* had a protein product. No peptides are detected for *HEPN1* in PeptideAtlas [22] and in the Human Protein Atlas [25] the antibody identification is tagged as uncertain.

**Figure 4.**
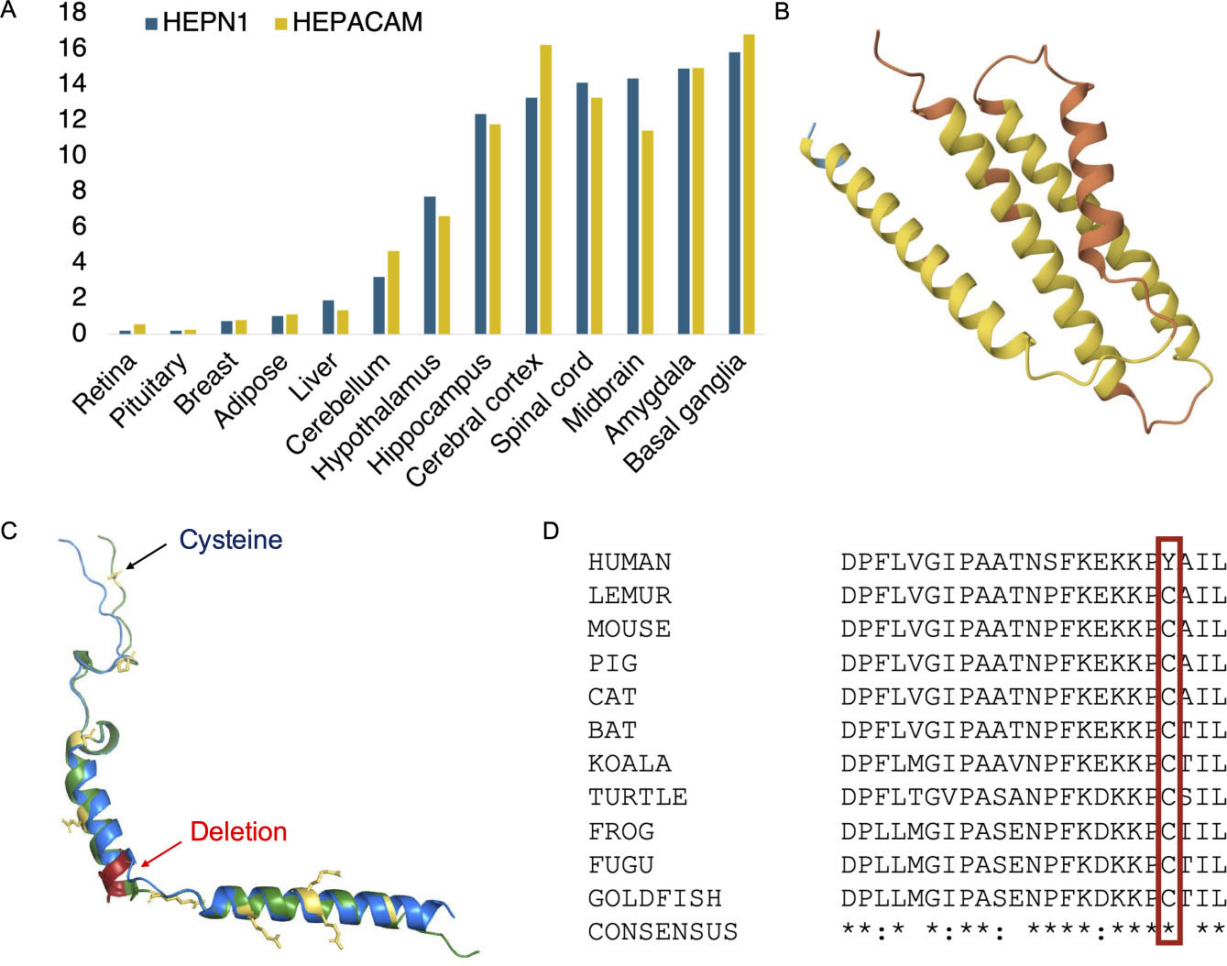
Potential non-coding genes *HEPN1, PBOV1*, and *GNG14*. Panel A shows the Human Protein Atlas transcript expression per tissue as a percentage of all the detected expression. *HEPN1* and *HEPACAM* have almost identical expression profiles. Panel B shows the predicted AlphaFold [43] structure for *PBOV1*. Although a four-helix bundle is predicted by AlphaFold, the reliability scores for this model are very poor. Regions coloured in yellow have AlphaFold reliability scores <70 and regions in orange <50. Panel C superimposes predicted AlphaFold structures of the human (blue) and cat (green) *GNG14* gene product. Residues in conserved positions that have mutated in the human protein are shown as yellow sticks; the deleted human region is mapped onto the predicted structure of cat *GNG14* in red. In panel D, an alignment of the C-terminal of vertebrate *GNG14* sequences. The human-specific tyrosine for cysteine swap is marked with a red box.

The predicted coding frame of *HEPN1* is not conserved beyond great apes because of a frameshift mutation. Despite this, *HEPN1* is annotated as a coding gene (using the wrong frame) in many primates and mammals. Meanwhile, the eight most common GNOMAD germline variants in *HEPN1* are non-synonymous or high impact [26]. The predicted human *HEPN1* isoform would have a long hydrophobic tail, so if translated would almost certainly be a candidate for *BAG6* ubiquitin-mediated degradation [33]. Most interestingly, *HEPN1* has an almost identical expression pattern to *HEPACAM* (Fig. 4A). The evidence suggests that *HEPN1* may be involved in regulation but is certainly not a coding gene and should probably be classed as a natural antisense transcript of *HEPACAM* [34].

*BLID* is a single exon gene discovered on the same chromosome and at more or less the same time as *HEPN1* [35]. Its identification as a protein coding gene was partly based on an imaginary BH3-like motif. If coding, *BLID* would be a human-specific protein coding gene because only the human ORF has an ATG and outside of great apes the ORF is broken by frame shifts. Although there are papers that ascribe functions including binding to *BCL2L1* [36] and regulation of the Akt pathway [37], it seems extremely unlikely that the Akt pathway—a pathway that is conserved across eukaryotes—would be controlled by a human-specific gene. As would be expected if this gene were not a coding gene, there is no evidence of purifying selection from germline variants [26], no peptides are detected for *BLID* in PeptideAtlas, and none of the Human Protein Atlas [25], FANTOM [24], or GTex [23] RNA datasets have transcription evidence for *BLID*, not even in breast tissue where it is supposed to be highly expressed [38]. Human Protein Atlas does record minimal transcript evidence for a small number of cancer cell lines. This lack of experimental evidence does suggest that *BLID* is most likely to be a non-coding cancer antigen. As a curiosity, one further feature that *BLID* has in common with *HEPN1* is that both have retracted papers that support functional roles [39, 40].

*PBOV1* is another single exon gene. There are papers with western blots that support the *PBOV1* protein in prostate and ovarian cancers [41, 42] and RNA support in lymphoma and leukaemia in the Human Protein Atlas. AlphaFold [43] predicts that a hypothetical *PBOV1* protein would fold into a four-helix bundle (Fig. 4B), though the prediction is low quality. Once again, *PBOV1* would be a human-specific coding gene because all primate species have frame shifts relative to humans. There is no hint of purifying selection to support any *de novo* function; the eight most common GNOMAD germline variants are non-synonymous or high impact. As with *BLID*, there are no peptides detected for *PBOV1* in PeptideAtlas and there is no protein or transcript support in normal tissues in Human Protein Atlas. Again, like *BLID, PBOV1* appears to be a cancer antigen rather than a standard coding gene. There are plenty of other potential non-coding genes that are likely cancer antigens, including *HMHB1, DCANP1*, and *PRAC2*. The human gene set also has several cancer antigen families that have little to no protein-level support. Although cancer antigens were once thought to be coding, most are now thought to be aberrant [44].

### Potential non-coding genes and pseudogenization

There are also potential non-coding genes in the intersection that do have an evolutionary history. One such case is *GNG14*, a two-exon gene that is clearly coding in most mammalian species. *GNG14* would produce a heterotrimeric G-protein gamma subunit, one of three proteins in a GTP-binding complex that mediates signal transduction across the plasma membrane [45]. There are 13 other G-protein gamma subunit isoforms annotated in the human genome.

Although the coding exons of *GNG14* transcripts are highly conserved across most mammals, the gene seems to have lost importance among monkeys because ~20% of transcripts have ORF-disabling mutations. Great apes have three radical mutations in positions that are otherwise highly conserved across all GNG proteins. Human *GNG14* also has a frameshift mutation close to the 5′ splice site of the second exon that can be skipped, but only at the cost of eliminating three of the most conserved amino acids in *GNG14* (Fig. 4C).

Finally, it is known that G-protein gamma isoforms are post-translationally modified at the C-terminal. The modified residue, a cysteine residue that is four amino acids from the C-terminus, is vital to the function of all G-protein gamma isoforms. The cysteine is prenylated, the final three amino acids are cleaved, and finally the prenylated cysteine is carboxymethylated [46]. The carboxymethylation is required for the association to the plasma membrane, and without it the complex cannot bind receptors or interact with downstream effector proteins [47, 48]. Any great ape *GNG14* isoform would not be able to perform any of these functions because this cysteine has been mutated to tyrosine (Fig. 4D).

If human *GNG14* does produce a protein, it would clearly not function as a G-protein gamma isoform. There is no evidence that the mutated isoform might be functionally important in any other role either—two of the three most common germline variants in GNOMAD are a stop gain and a start lost variant. Again, there is no protein or transcript evidence for *GNG14*, and it has no supporting publications. *GNG14* is quite clearly a pseudogene in great apes, and probably also in monkeys.

Connexins are four transmembrane helix bundles that associate to form gap junction channels that allow electrical signal transfer and metabolite diffusion between cells [49]. There are >20 connexin isoforms annotated in the human genome. One of these, *GJE1*, has just four extra-cellular cysteines instead of the usual six, and mouse experiments have shown that it does not form gap junctions [50]. *GJE1* is clearly conserved across vertebrates, but conservation evidence shows just as clearly that *GJE1* is a pseudogene across primates. Despite this, human, chimpanzee, and gorilla *GJE1* transcripts have intact ORFs, so *GJE1* is annotated as a coding gene in these species.

Is *GJE1* coding in humans, chimpanzees, and gorillas? Human *GJE1* is not supported by experimental evidence in PeptideAtlas or the Human Protein Atlas. Gorilla *GJE1* has lost one of the four remaining conserved extracellular cysteines. Given the conservation patterns across primates, *GJE1* probably pseudogenized at least 80 million years ago. So, this raises the question, which is more unlikely, that gorilla, chimpanzee and human maintained a *GJE1* coding gene without experimental support, or that *GJE1* pseudogenized in the ancestor of primates yet the ORF has been maintained over 80 million years?

**Figure 5.**
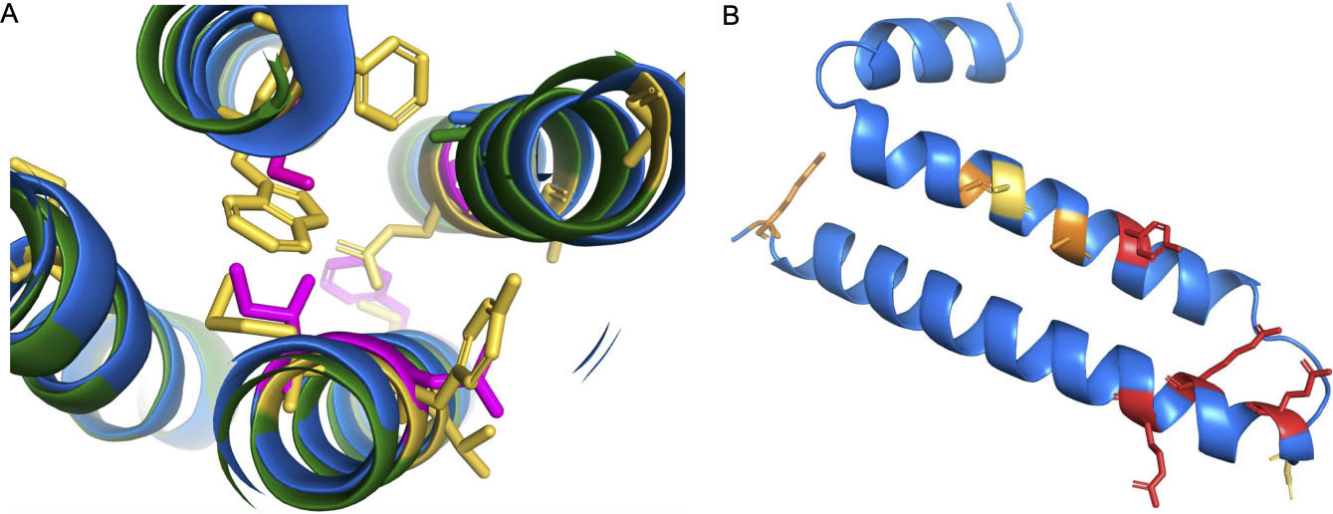
Potential non-coding genes *GJE1* and *HIGD2B*. Panel A shows a cross-section of the predicted AlphaFold structures of human (blue) and cat (green) *GJE1*. Cat *GJE1* conserved residues that have mutated in the human sequence are shown as yellow sticks; some of the mutated human residues are shown in magenta. Cat *GJE1* forms a four-helix transmembrane bundle, but many of the human mutations would adversely affect the packing of the transmembrane helices. From top, clockwise, tryptophan (cat) to serine (human), glutamate to alanine, tyrosine to asparagine, methionine to isoleucine, and in the background, cysteine to phenylalanine. The tryptophan to serine mutation would require the human sequence to use the fictitious 5′ splice site. In panel B, the predicted AlphaFold structure of human *HIGD2A*. Residues in conserved positions that have mutated in the human *HIGD2B* sequence are shown as sticks; sticks are coloured red when the mutation is in a wholly conserved position and orange when the mutation is in an almost completely conserved position, and yellow when it is another radically different amino acid in a less conserved position.

Fortunately, there are clues that can be used to come to a decision. Firstly, humans, chimpanzees, and gorillas have radical amino acid changes [51] in conserved positions that will almost adversely affect the packing of the transmembrane helices (Fig. 5A). Secondly, in humans, chimpanzees, and gorillas, the 5' end of the third coding exon of *GJE1* has had to be shortened by four codons in order to escape a stop codon that is unique to the three species.

This shortening of the exon provides conclusive evidence. To make up for the loss of four amino acids in the protein from the 5′ end of the third coding exon, the 3′ end of the second coding exon has been extended by three codons downstream. This mutation is possible and might produce a functional protein without too much loss of function since the swap occurs at the end of an exon. However, the predicted structural model suggests that the swap would adversely affect the packing between two transmembrane helices (Fig. 5A). In addition, there is no experimental evidence to support the use of the novel splice site (the cDNA from RefSeq is an inferred model), and the novel 5′ intronic splice acceptor site would be GTGTGC, which is not an accepted splice acceptor pattern [52]. If the novel 5′ intronic splice acceptor site is not unsupported, the *GJE1* ORF has lost four important amino acids in humans, chimpanzees, and gorillas.

The jumped stop codon in the final exon, the radical amino acid changes that would affect the packing of the transmembrane helices (Fig. 5A), and the lack of experimental support for the inferred novel splice site is compelling evidence that *GJE1* is just as much of a pseudogene in humans, chimpanzees, and gorillas as it is in all other primate species.

One final example of potential non-coding genes annotated by all three reference databases is the gene *HIGD2B. HIGD2B* was identified by both Clamp *et al*. [9] and Church *et al*. [10] as a pseudogene and has now been tagged as a potential non-coding gene in all three of our analyses [12, 14]. It duplicated from *HIGD2A*, an ancient, conserved gene involved in the stabilization of the cytochrome *c* oxidase (complex IV) in the mitochondrial membrane [53], via retrotransposition at the base of catarrhini. The loss of *HIGD2A* has been shown to result in a damaged complex IV that lacks *COX3* [54]. *HIGD2A* has widespread expression, but *HIGD2B* is expressed only in testis.

*HIGD2B* is annotated as coding because the ORF is intact and because there is plenty of transcript evidence in testis. Despite that, and despite the large number of testis tissue experiments in PeptideAtlas, there is no supporting protein evidence at all. Although *HIGD2B* has undergone substantial changes in sequence since retrotransposition and could conceivably have gained a new function, evidence from cross-species alignments shows that any novel function was obviously not important enough to stop it becoming a pseudogene across all non-ape old world monkeys and in orangutans.

As with *GJE1*, many of the amino acid changes in human *HIGD2B* are radical [51]. We generated an alignment of 35 vertebrate *HIGD2A* proteins that has 29 completely conserved amino acid positions (Supplementary Fig. 2). Four of these positions, along with another 3 positions that are almost completely conserved, have different amino acids in *HIGD2B* proteins (Fig. 4B). Three of these changes would be radical. In addition, gorilla *HIGD2B* has two more radical amino acid changes and gibbon *HIGD2B* has another three. The changes are such in gorillas that AlphaFold predicts that the second membrane spanning helix will be four residues shorter.

These radical changes, the widespread pseudogenization of *HIGD2B* across monkeys, and a complete lack of protein evidence in all tissues (including testis) in PeptideAtlas and Human Protein Atlas strongly suggest that *HIGD2B* is another pseudogene that has not yet been affected by an ORF-breaking mutation.

### Not all non-coding genes are tagged as potential non-coding

**Figure 6.**
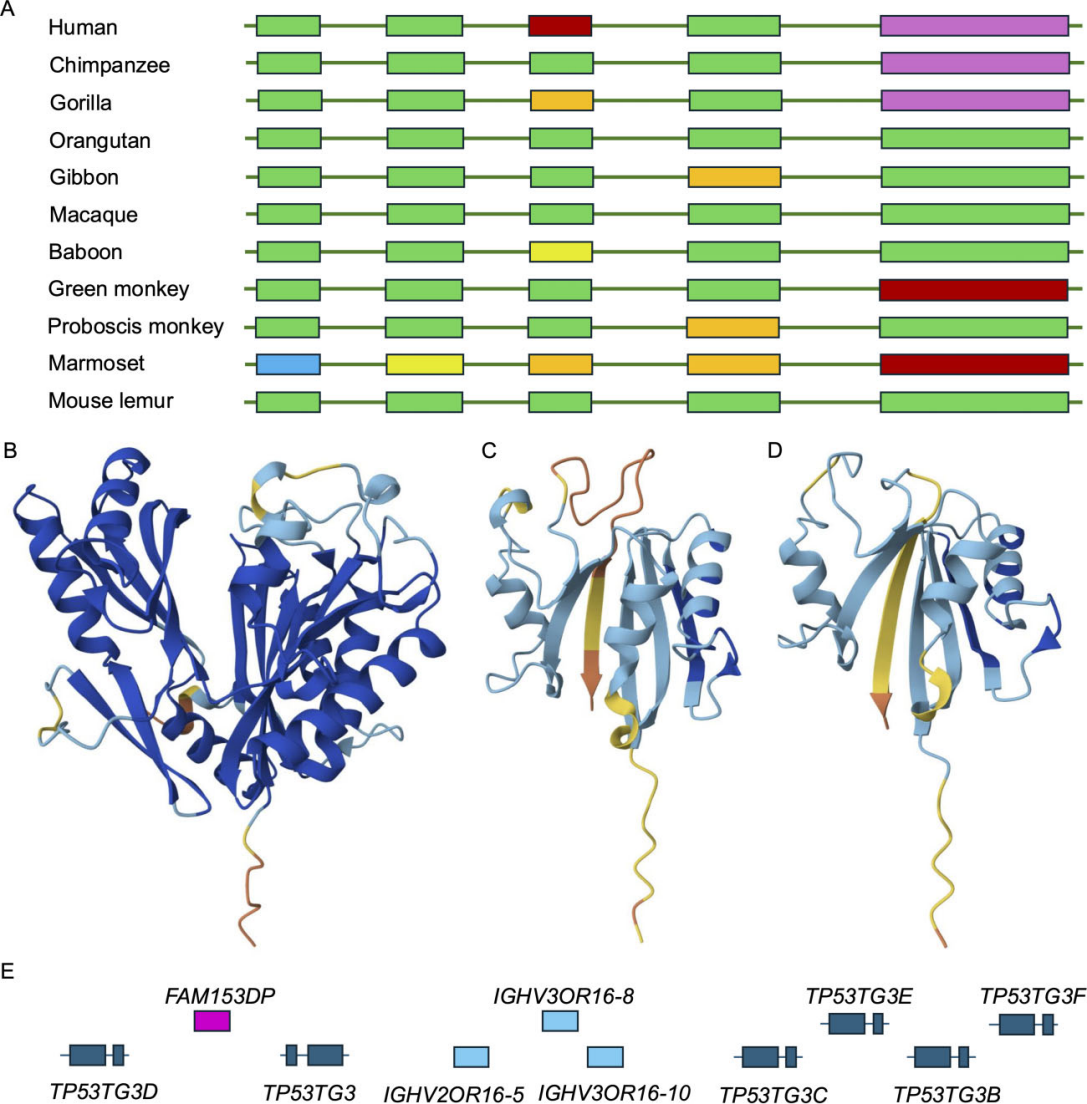
Potential non-coding genes *FTCDNL1* and the *TP53TG3* family. Panel A shows the exon structure of primate *FTCDNL1* genes and pseudogenes. Exons and introns not to scale. Exons missing the ATG are in blue, exons missing splice sites in yellow, exons with frameshift mutations in orange, exons with stop codons in red, and missing exons in purple. Panels B–D show AlpahFold models of *FTCDNL1*. All are coloured by AlphaFold reliability score, dark blue indicates that the region has a score of >90, light blue shows that the score is between 70 and 90, yellow <70, and orange <50. (B) The predicted structure of cow *FTCDNL1* with both lobes of the structure. (C) The predicted structure of the UniProtKB reference sequence for human *FTCDNL1*, a beta-sheet structure in which one of the integral strands has a very low reliability score because it is made up of a string of five phenylalanine residues that have had to be shoehorned into position. Panel D shows the predicted structure of the MANE Select reference sequence [15] for human *FTCDNL1*. As with the UniProtKB reference sequence, it is also missing a lobe of the structure, and again the final strand integral to the protein has a very low reliability score. The APPRIS principal isoform [21] is different again from the two reference proteins shown here, but the model is not any better. Panel E shows the section of chromosome 16 that houses most of the *TP53TG3* orthologues. Exons are not to scale.

There are also genes in the intersection of the three reference sets that are not tagged as potential non-coding, but that are highly likely to be misclassified as coding. We have already detailed two genes in previous papers; neither *WASHC1* [8] nor *PLK5* [14] is tagged as potential non-coding in this analysis. Another obvious case and not tagged as potential non-coding is *FTCDNL1*, an uncharacterized five-exon gene related to osteoporosis [55]. It is an ancient gene with clear coding conservation across mammals, but conservation across primates is patchy (Fig. 6A). It seems to be conserved in lemurs but is clearly no longer coding in new world monkeys; in marmosets all five coding exons have high-impact mutations. Among old world monkeys, it is less obvious whether *FTCDNL1* is still a coding gene, but in humans, chimpanzees, and gorillas it has almost certainly become a pseudogene because all four species have lost the final coding exon. In addition to the lost exon and stop codon, gorilla *FTCDNL1* has a unique frameshift mutation and human *FTCDNL1* has a unique stop codon in the third exon.

Mammalian *FTCDNL1* proteins fold into a single globular domain (Fig. 6B), but human *FTCDNL1* isoforms are either shortened by the human-specific stop codon in exon 3 or produced from transcripts that skip exon 3 and terminate in non-conserved regions downstream of exon 4. The loss of exons 3 and 5 would lead to a radically changed protein structure (Fig. 6C and D). Although *FTCDNL1* seems to be expressed in almost all tissues at the RNA level, there is no evidence that it is translated into protein in PeptideAtlas. Human Protein Atlas has no protein evidence in tissues but does find antibody evidence for the isoform translated from the MANE Select transcript in vesicles in cell lines. This is a curious result, given that the *FTCDNL1* protein does not have a signal peptide. In any case, the UniProtKB *FTCDNL1* isoform (just like the predicted isoform of *HEPN1*) has a long hydrophobic tail, so if translated would almost certainly be a candidate for *BAG6* ubiquitin-mediated degradation [33].

Gene *TP53TG3* was discovered in an analysis of a cancer colon cell line a quarter of a century ago [56]. However, no further work has ever been carried out on this gene or any of its paralogues. There are five sequence-identical coding paralogues of *TP53TG3* and two pseudogenes found on the short arm of chromosome 16 (Fig. 6E). *TP53TG3* genes were all tagged as potential non-coding in our previous analysis [14], but not in this investigation because UniProtKB annotates them as having homology support and this feature is not considered to be potential non-coding in this study. It is not clear which homologous proteins UniProtKB used as evidence, since there are none.

*TP53TG3* genes are a very recent innovation. There are conserved ORFs in only chimpanzees and gorillas. Gorillas have one copy of the gene and a pseudogene, and chimpanzees and bonobos have a pseudogene and several *TP53TG3* genes. Beyond these species, *TP53TG3* genes cannot be coding because of the multiple frame shifts and stop codons that break the ORF and because the splice sites in the various transcripts are not conserved either. Given that, it seems highly unlikely that any of the *TP53TG3* paralogues play any role at all in the important cell cycle checkpoint pathway as was originally predicted [56].

There is substantial RNA evidence for *TP53TG3* and its paralogues in testis and epididymis in Human Protein Atlas, and there is also some in cancer cell lines. Despite the substantial RNA evidence in these tissues, there is no peptide evidence in PeptideAtlas. Given the transcript expression, there is no obvious explanation for not detecting peptide evidence in one of the many large-scale testis tissue experiments in PeptideAtlas, especially since, according to PeptideAtlas, the protein has multiple detectable tryptic peptides and it is known that constructs of *TP53TG3* have a long half-life [56]. Given the lack of conservation and lack of evidence for any protein, it would be remarkable if any of these six genes were protein coding.

## Conclusions

We have carried out a follow-up of our in-depth analysis of the coding genes annotated in three main human reference databases [14]. The human reference gene count currently stands at somewhere between 19 950 and 20 485 coding genes depending on which of the three main references is used. When merged the three gene sets contain 21 871 coding genes, the three gene sets agree on 19 268 coding genes, and there are 2603 genes that the three reference sets do not agree on.

When readthrough genes and immunoglobulin fragments are omitted from the sets, we show that the number of genes agreed upon by all three reference annotators has increased by 249 since we last compared the three annotations in 2018 [14]. Under these conditions, the number of coding genes that the reference databases disagree on falls to slightly over 1500 with the Ensembl/GENCODE reference set standing out because it has only 112 coding genes that are not also coding in RefSeq and UniProtKB. This demonstrates that the MANE and GIFTS projects that Ensembl/GENCODE shared with RefSeq and UniProtKB have been effective.

Omitting readthrough genes from the sets is not just a theoretical exercise, since Ensembl/GENCODE has plans to reclassify all readthrough genes that are currently classified as coding. Currently, Ensembl/GENCODE annotates 655 readthrough genes as protein coding. We find a peptide for just one of these genes and there is evidence to suggest that the exon it maps to is really part of one of the parent genes. The three gene sets are substantially further from a consensus while readthrough genes are still annotated as coding. Readthrough genes are of no biological interest and only serve to add noise and to complicate large-scale analyses unnecessarily. Many users of the reference gene sets are unaware of the effect of readthrough genes on their analyses.

Readthrough genes were one of the eight potential non-coding features that we used to flag potential non-coding genes in the Ensembl/GENCODE gene set. In this analysis, there were fewer potential non-coding features than in our previous analyses [12, 14], so it was not surprising there were fewer potential non-coding genes here. As is clear from the peptide evidence, not all of the 1121 Ensembl/GENCODE genes we flag as potential non-coding genes will turn out to be non-coding, but many will be reclassified. A total of 379 coding genes were reclassified as not coding after the previous analysis [14].

We could also predict potential non-coding for the other two reference sets. We flagged 943 UniProtKB genes and 706 as RefSeq genes as potential non-coding even though not all features applied to these gene sets. Most of the potential non-coding genes were outside of the intersection between the three sets, more than two thirds of UniProtKB outside of the intersection (668) were tagged as potential non-coding, and 72% of Ensembl/GENCODE genes (846 of 1 175). In the case of the 681 RefSeq genes outside of the intersection, germline variation data showed that a large majority of these genes are not under purifying selection.

We believe that almost all of the 2603 predicted coding genes beyond the intersection between the three sets are likely to be non-coding genes or pseudogenes, though there will certainly be some exceptions as we recently have shown [57, 58]. In addition, there are 275 potential non-coding genes in the intersection of the three catalogues. Together with the 2603 genes outside of the intersection, the potential non-coding genes in the intersection mean that the reference catalogues contain as many as 2878 coding genes that are potentially pseudogenes or non-coding genes.

Determining whether a gene is coding or not is not always a simple process. Some genes may have published functional evidence, which may complicate what would otherwise be a simple decision [59, 60], while other genes might be supported by evidence from large-scale databases such as Human Peptide Atlas antibodies or PeptideAtlas peptides that need to be contrasted to see whether they are reliable. Pseudogenes and coding genes are particularly difficult to disentangle if the ORF is maintained but has no supporting evidence.

However, as we have shown here and in an earlier analysis [8], it is not impossible. Here, we were able to use experimental information to show that at least 25 genes inside the intersection are almost certainly not protein coding genes. At the same time as we have shown that a number of coding genes are likely to be pseudogenes, we have also shown that several genes that were previously thought to be pseudogenes are in fact coding despite having broken ORFs [57, 58].

One particularly problematic set of genes are those large recently duplicated gene families such as the PRAMEF family [61] and the USP17L family [62]. These families have multiple recently duplicated genes and little to no supporting evidence, and it is not clear whether one, none, or all these genes are coding. The SINE-Alu-derived NPIPB family [63], for instance, does have some supporting evidence [64], but here it is not obvious whether this evidence supports one or two of the members of the family or all of them. If just one or two of these genes are coding, they are so similar that it is not clear which genes are coding. Curators usually annotate all genes of recently duplicated families as coding as long as they have intact ORFs.

Conversely, cancer antigens, a set of genes that previously generated doubts about their coding status, are much more clear-cut. These ORFs may produce proteins [58] under certain circumstances, but there is increasing evidence that cancers generate much aberrant translation [44]. These genes should not be classified as coding because their presence in the reference set of coding genes can only complicate biomedical experiments. Cancer antigens should not be annotated as protein coding genes and instead should be annotated as what they are: cancer antigens.

This work is an analysis of the reference coding gene set from the GRCh38 assembly of the human genome. The annotation of both the new CHM13-T2T assembly [65, 66] and the human pangenome [67] has the potential to add to the gene numbers here. The CHM13 assembly completed the remaining 8% of the human genome and unearthed 140 novel genes with protein coding gene pedigree [65]. We found supporting evidence for two novel protein coding genes in the CHM13-T2T assembly [8], but most of the 140 genes were from large, recently duplicated families. For example, 31 of the new genes are duplications of *TAF11L5* and 34 are most similar to the USP17L genes. The new CHM13-T2T assembly is a hugely important achievement for many reasons, but it now seems unlikely that it will add more than a handful of new coding genes to those already known. The only source that might add a substantial number of novel coding genes would be conserved short ORFs found in untranslated regions [15, 68], though many of these may be alternative transcripts rather than novel genes.

In this analysis, we have shown that annotators have made good progress with the unification of the three reference human gene sets, but that the number of coding genes annotated across these sets is still inflated. The three main reference databases annotate at least 2000 coding genes that will either never produce proteins or only produce proteins in dysregulated conditions such as cancers. Without these genes the human reference coding gene set will have close to 19 000 coding genes [12, 14]. Determining an agreed-upon set of reference coding genes is of fundamental importance to researchers and we hope that this work will inspire the three main reference databases, and perhaps others [69], to continue to work together towards a final agreed set of *bona fide* coding genes.

## Supplementary Material

baaf045_Supplemental_Files

## Data Availability

The data underlying this article are available in the following public repositories: RefSeq, https://www.ncbi.nlm.nih.gov/datasets/gene/taxon/9606/; UniProtKB, https://www.uniprot.org/proteomes/UP000005640; Ensembl/GENCODE, https://www.gencodegenes.org/human/; and PeptideAtlas, https://peptideatlas.org/ The merged list of genes and related data are available in the Supplementary Material (Supplementary Table 1) and will be available in the APPRIS database (https://appris.bioinfo.cnio.es/).
