# Supplementary material for: The state of the human coding gene catalogues
Source: Database (Oxford). 2025 Sep 24;2025:baaf045. doi: 10.1093/database/baaf045 (PMC12462614; doi:10.1093/database/baaf045)
Supplement: baaf045_Supplemental_Files [file baaf045_supplemental_files.zip › Supplementary.docx]

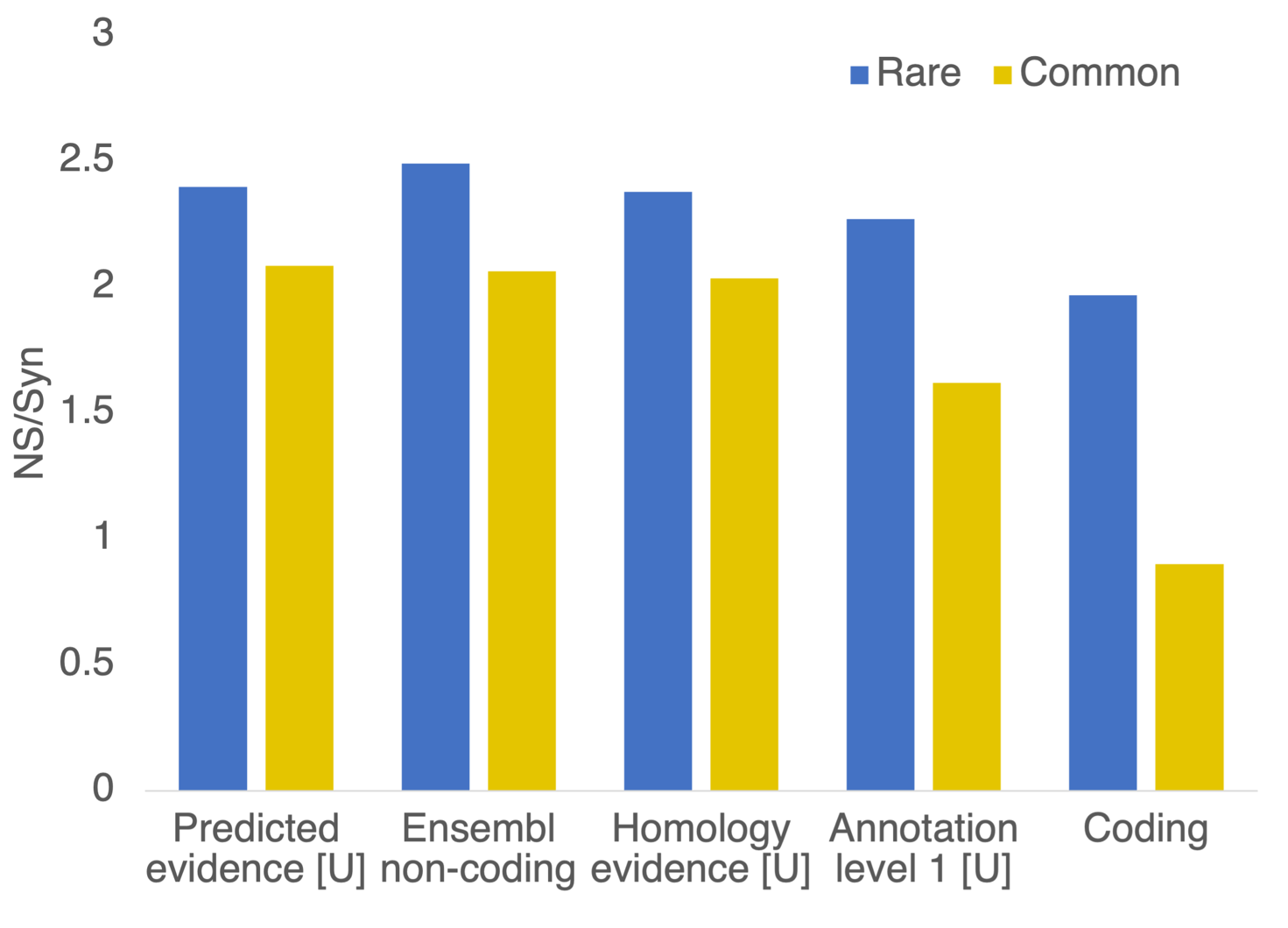


**Supplementary Figure 1**

The NS/Syn ratios calculated for potential non-coding features excluded from the analysis. Features marked with a “U” are annotator evidence codes that come directly from the UniProtKB database. Ensembl non-coding genes are those marked as non-coding, non-functiona or pseudogene. Coding gene NS/Syn ratios (from genes under purifying selection) are included as a control.

**HIG2DB HUMAN MA-----------TLGFVTPEAPFESSKPPIFEGLSPTVY-S-NPEGFKEKFLRKTRENP**

MARMOSET MA-----------TPGPVTPEASLEPSKPPVIEGFSPTLY-S-KPESFKEKFIRKVRENP

MACAQUE MA-----------TPGPVIPEVPFEPSKPPVIEGLSPTVY-R-DPETFKEKFLRKTRENP

**HIG2DA HUMAN MA-----------TPGPVIPEVPFEPSKPPVIEGLSPTVY-R-NPESFKEKFVRKTRENP**

RAT MA-----------APGPVSPEAPFDPSKAPVIEGFSPTVY-T-NPEGFKEKFIRKTRENP

MOUSE MA-----------APGPVSPEAPFDPSKPPVIEGFSPTVY-S-NPEGFKEKFIRKTRENP

HAMSTER MA-----------APRPVSSEAPFDPSQTPVIEGFTPTVY-S-NPESFKDKFIRKTRENP

BAT MT-----------TPGPVTPGTPFEPSQPPVVEGISPSVY-S-TTESFKEKFLRKTRENP

GUINEA PIG MA-----------TPGPVTPEAPFKPSQPPVIEGFNPSVH-I-HQEGFKEKFLRKTRENP

PIG MA-----------TPGPATPEAPFEPSHPPVIEGFSPTVY-S-TSESFKKKFIRKTRENP

TARSIER MA-----------TPGPVTPEVPFEPSQPPVIEGFSPSVY-G-NPESFKEKFLRKTRENP

CAT MA-----------VPGPVTPEAPFEPSQPPVIEGFSPSVY-S-TPESFKEKFLRKTRENP

DOG MA-----------APGPVTPGAPFEPSQPPVIEGFSPSVY-S-PPESFKEKFLRKTRENP

FOX MA-----------APGPVTPGAPFEPSQPPVIEGFSPSIY-S-TPESFKEKFLRKTRENP

POLAR BEAR MA-----------APGPVTPGAPFEPSQPPVIEGFSPSIY-N-TQESFKEKFLRKTRENP

SEAL MA-----------APGPVTPGAPFEPSQPPVIEGFSPSIY-S-TQESFKEKFLRKTRENP

DOLPHIN MA-----------TPGPVTPEAPFEPSQPPVIEGFSPSVY-S-TSESFKEKFLRKTRENP

SHEEP ME-----------TPGRVTPEAPFEPSQPPVIEGFSPSVY-S-TSEGFKEKFIRKTRENP

COW ME-----------TPGRVTPEAPFEPSQPPVIEGFSPSVY-S-TSESFKEKFIRKTRENP

KILLFISH MAATTRPSEPESHVKEQPPGALPFVLSQPPVIEGFRQSPK-V-KDETFKEKFIRKTKENP

SNAILFISH MA-GATAVSELTPAAKSPAAFAPFDFSQPPDIEGFSRLPA-A-RDETFKEKFLRKTKENP

TROPICAL FROG --------------------------MAHPEIEGFTPSST-Y-GDEGFKSKFIRKVKENP

AFRICAN FROG --------------------------MAQPEIEGFTPS-T-Y-TSEGFKGKFIRKVKENP

SHARK ---------------MAAPVREEVSLSKPPVIEGFHPAPR-P-REESFRDKFIRKTSENP

COD MAAASTPAVVNSPI-TQQSGGMPFDFSKPPVIEGFTPVSR-R-KDETFKEKLLRKTKENP

TROUT MAAATTPVVPEQSASTT--HLPMLDISKPPVIDGFTPLSR-P-REETFQEKFMRKSKENP

CAVE FISH MAVASSAVQEERAGRTALPGAAAFNPGSPPVIEGFTPLPR-Q-KEESFKEKFLRKTKENP

ZEBRAFISH MATAAAPVSPDQPGKSA-SPPVLLDLSQPPVIEGFSPTSR-T-REEGFKDKFIRKTKENP

CATFISH MAAASSRVEQASPVKTAPPIAAGFDLSDPPVIDGFTPLSR-P-REEGFKDKFIRKTKENP

HERRING MAASPTVVSQDQAANAASQGPIPFDISKPPIIEGFTPLPR-H-REEGFKDKFIRKTKENP

MILKFISH MAAASTPVERDQVVKTPQSGPVPFDISKPPVIEGFTPLPR-A-KEEGFKDKFIRKTKENP

CHICKEN ---------------MAAGPPPPLEPIPLPVY-----------RDEGFADKFRRKTRENP

ZEBRA FINCH ---------------MAAGPPPPLEPSPLPTF-----------PEEGFTEKFVRKTRENP

BARN SWALLOW ---------------MAAGPPPPLDPIPLPTF-----------TEEGFAEKFLRKTRENP

WALL LIZARD ---------------MAQSAPPPFDPNNPPLIEGFTPTAY-H-PEEGFGDKFRRKTRENP

ANOLE LIZARD ---------------MTQAPPPPFDPSRPPLIEGFQPGAF-QRREEGFADKFLRKTRENP

* * * *: ** ***

**HIG2DB HUMAN VVPIGFLCTAAVLTNGLYCFHQGNSQCSRLMMHTQIAAQGFTIAAILLGLAATAMKSPP-**

MARMOSET MVPIGCLATATALGYGLYCFHKGHSRRSQLMMRTRIAAQGFTIAAILVGLGVTSMKSRP-

MACAQUE VVPIGCLATVAALTYGVYSFYRGDSRRSQLMMRTRIAAQGFTVTALLLGLAVTAMKSRP-

**HIG2DA HUMAN VVPIGCLATAAALTYGLYSFHRGNSQRSQLMMRTRIAAQGFTVAAILLGLAVTAMKSRP-**

RAT MVPIGCLGTAAALTYGLYCFHRGQSHRSQLMMRTRIAAQGFTVVAILLGLAASTMKSRS-

MOUSE MVPIGCLGTAAALTYGLYCFHRGQSHRSQLMMRTRIAAQGFTVVAILLGLAASAMKSQA-

HAMSTER MVPIGCLGTAAALSYGLYCFHRGQSHRSQIMMRTRIAAQGFTVVAILLGLAASAMKSRS-

BAT MVPLGCLSTAAALTYGLYCFHRGQSQRSQLMMRTRIAAQGFTIVAILVGLAASALKSRP-

GUINEA PIG MVPIGCLGTAAALTYGLYCFHQGHSQRSQFMMRTRIAAQGFTVAAILLGLAASAMKSRS-

PIG MVPIGCLGTASALTYGLYCFHRGQSQRSQLMMRTRIAAQGFTIVVILVGLAASTMRSRP-

TARSIER MVPIGCLGTAAALTYGLYCFHRGHSQRSQLMMRTRIAAQGFTVAAILLGLAASALKSRP-

CAT MVPIGCLGTAAALTYGLYCFHRGQSHRSQLMMRTRIAAQGFTVAAILLGLAASAMRSRS-

DOG MVPVGCLGTAAALTYGLYCFHRGQSHRSQLMMRTRIAAQGFTVAAILLGLAASAMKSRS-

FOX MVPIGCLGTAAALTYGLYCFHRGQSHRSQLMMRTRIAAQGFTVAAILLGLAASAMKSRS-

POLAR BEAR MVPIGCLGTAAALTYGLYCFHRGQSHRSQLMMRTRIAAQGFTVAAILLGLAASAMKSRS-

SEAL MVPIGCLGTAAALTYGLYCFHRGQSHRSQLMMRTRIAAQGFTVAAVLLGLAASAMKSRS-

DOLPHIN MVPIGCLGTAAALTYGLYCFHRGQSQRSQLMMRTRIAAQGFTVVAILMGLAASTMKSRP-

SHEEP LVPIGCLGTAAALTYGLYCFHRGQSQRSQLMMRTRIAAQGFTIVAILVGLAASTLKSRP-

COW LVPIGCLGTAAALTYGLYCFHRGQSQRSQLMMRTRIAAQGFTIVAILVGLAASTLKSRP-

KILLFISH FVPIGCLGTTGMLMYGLRSFHQGKTKQSQMFMRGRIFAQGFTVVAIIVGIFATALKPKQ-

SNAILFISH FVPIGCLGTAGALVYGLRAFNQGKTRQSQLMMRGRIFAQGFTVVAIIAGVFITAMKPKQ-

TROPICAL FROG FVPIGCLATAGALTYGLISFKQGKTQQSQLLMRTRILAQGFTVAAIMFGVVMTAMKPRIT

AFRICAN FROG FVPIGCLATAGALTYGLISFKQGKTRQSQLLMRTRILAQGFTVAAIMVGVVMTALKPSET

SHARK FVPLGMLGTAGALTYGLIAFNHGKTRHSQLSMRARIFAQGFTIVAIVVGVVATTLKPK–

COD FVPIGCLGTAGALAYGLRAFHQGKTRQSQMLMRGRIFAQGFTVFAIIFGVVATQLKPKQ-

TROUT FVPIGCLGTAGALMYGLRAFKQGKTRQSQLLMRGRIFAQGFTVVAIIFGVFTTALKKD--

CAVE FISH FVPIGCLGTAGALTYGLRAFKHGKTHQSQMLMRTRIFAQGFTVIAIIVGVAATALKSKQ-

ZEBRAFISH FVPIGCLGTAGALIYGLGAFKQGKTRQSQLLMRTRIFAQGFTVVAIIVGVAATALKAKP-

CATFISH FVPIGCLGTAGALIYGLRAFKQGKTRQSQLLMRTRIFAQGFTVVAIIVGVAAAALKPRQ-

HERRING FVPIGCLGTAGALIYGLRAFKMGKTRQSQLSMRMRIFAQGFTVVAIIVGVASTALKPKQ-

MILKFISH FVPIGCLGTAGALIYGLSAFRQGKTRQSQLLMRARIFAQGFTVVAIIVGVATTALKSK–

CHICKEN LVPLGCLCTLGVLTYGLISFKRGNTRHSQLMMRARVVAQGFTVAALLGGMVATALRARS-

ZEBRA FINCH LVPLGCLCTVSVLVYGIICFKRGQTRRSQLMMRARVIAQGCTFAALLGGMVATALKSRQ-

BARN SWALLOW MVPLGCLCTVGVLAYGVICFKKGNTRRSQLMMRARVVAQGFTIASVVGGMMATAIRSRQ-

WALL LIZARD LVPIGCLGTAGVLAYGLICFKKGNTLQSQRMMRARVLAQGFTVAAILVGVVVASMKPKK-

ANOLE LIZARD LVPVGCLGTAGVLTYGLICFKRGNTHQSQIMMRARILAQGFTVAALVVGVVVTALKPKK-

.**:* * * * **: .* *. ** ** *: *** *. :: *: :

**Supplementary Figure 2. Alignment of human *HIGD2B* with vertebrate *HIGD2A* homologues**

Alignment of human *HIG2DB* with 35 vertebrate *HIGD2A* paralogues. *HIGD2A* sequence is shown in bold with the 29 residues that are completely conserved across the 35 *HIGD2A* in blue. *HIGD2B* sequence is shown in bold with residues that are different from completely conserved *HIGD2A* residues in red, residues that are radically different from highly conserved *HIGD2A* residues in orange and other residues that are radically different in green.
